# Determination of the Optimum Excitation Wavelength for the Parathyroid Gland Using a Near-Infrared Camera

**DOI:** 10.3389/fsurg.2020.619859

**Published:** 2021-01-21

**Authors:** Isao Tabei, Azusa Fuke, Astushi Fushimi, Hiroshi Takeyama

**Affiliations:** ^1^Department of Surgery, Daisan Hospital, The Jikei University School of Medicine, Tokyo, Japan; ^2^Breast, Thyroid, and Endocrine Division, Department of Surgery, The Jikei University School of Medicine, Tokyo, Japan

**Keywords:** parathyroid gland, near-infrared camera, excitation wavelength, autofluorescence, ICG

## Abstract

When performing thyroid/parathyroid surgery, difficulty detecting the parathyroid gland is a common experience because it is frequently mistaken with surrounding structures, including the thyroid gland, lymph nodes, and fat. To obtain successful surgical results, the auto fluorescent property of the parathyroid gland occurring at 820–830 nm has been used. Intraoperative visualization and detection by fluorescence enable protection of the gland from damage and unintended removal. Use of a near-infrared (NIR) camera has been proposed to indicate the parathyroid gland, but the devices and success rates have varied. This study aimed to define optimum excitation wavelength (EWL) by measuring the EWL of the parathyroid gland for its autofluorescence. Glands were exposed to EWL at 10-nm intervals from 670–790 nm with a light-emitting diode monochromator; autofluorescence intensity was recorded with a conventional NIR video camera. Autofluorescence intensity curves of three normal parathyroid glands were depicted; the optimum EWL was measured as 760–770 nm. Also, the illumination of the surrounding structures were compared at the optimum EWL. The auto fluorescent intensity of the parathyroid gland was 2-fold greater than for surrounding structures. This difference in fluorescence intensity should enable distinction of the parathyroid gland from surrounding structures. The clarification of the optimum EWL can guide refinements of the NIR camera for better surgical outcomes by improving detection of the parathyroid glands. Also, an understanding of optimum EWL should lead to developments for microscopic devices to unravel the still unknown mechanisms of the intrinsic autofluorescence of the parathyroid gland.

## Introduction

The parathyroid gland varies in number and anatomic location because of its embryologic characteristics (1). Surgeons often experience difficulty in detection of the parathyroid gland during thyroid/parathyroid surgery because it is frequently mistaken with surrounding structures, including the thyroid gland, lymph nodes, and fat (2). The near-infrared (NIR) camera with the indocyanine green (ICG) method is widely used for detection of the sentinel lymph node in early breast cancer surgery (3) and for evaluation of bloodstream efficacy and skin flap in breast reconstruction surgery. Also, the NIR camera has proven useful in vascular anastomosis in reconstructive surgery and for reducing anastomosis leakage in gastrointestinal surgery as well (4, 5). This approach is based on the property of ICG as a fluorophore when binding to albumin. This technique has also been introduced to detect the parathyroid gland during surgery (6–8). In 2011 Paras et al. first reported detection of the peak autofluorescence for the parathyroid gland occurring at 820–830 nm, using a laser diode at 785 nm to excite the gland (9). The fluorescence was 2- to 11-fold stronger compared with the surrounding structures. Numerous studies since 2011 have proposed this use of the NIR camera to indicate the parathyroid gland by autofluorescence, but the success rates and the devices used have varied (10–13). The choice of devices may be variable because the optimum excitation wavelength (EWL) for the parathyroid gland has not yet been defined; in addition, the mechanism for autofluorescence of the parathyroid gland has not yet been clarified. To date, the reason for the fluorophore as an intrinsic characteristic of the parathyroid gland remains unknown. To elucidate this issue, this study aimed to measure the EWL of the surgically extracted parathyroid gland for its autofluorescence to define the optimum EWL. To the best of the authors' knowledge, this report is the first to measure the optimum EWL of autofluorescence illumination for the parathyroid gland. Also, the illumination of the surrounding structures (thyroid, lymph node, and fat), which are often misidentified in the detection of the parathyroid gland, were compared to the parathyroid gland at the optimum EWL using a light-emitting diode (LED) light source for optic visual identification.

## Materials and Methods

### Measurement of the Optimum EWL for the Parathyroid Gland

Three parathyroid glands were collected from two patients who volunteered for monitoring of the extracted parathyroid glands by an NIR camera during thyroid surgery for measurement of the optimal EWL. The surgeries were performed at the authors' institution in April 2019. The only criterion applied was the availability of monitoring a normal parathyroid gland, so all preoperative measurements concerning the parathyroid were within normal. Gland samples 1 and 2 were the superior and inferior parathyroid glands, retrieved from a thyroidectomy for thyroid cancer of the left lobe. Sample 1 (superior gland) was extracted for possible involvement of the carcinoma. Sample 3 was an inferior parathyroid gland embedded within the lower pole of the thyroid obtained from a left lobe thyroidectomy performed for a patient with an oversized (6-cm) adenomatous goiter. Only one parathyroid gland was obtained from this operation because the superior parathyroid gland was preserved and not extracted. The parathyroid glands were exposed to EWL alternating at 10-nm intervals from 670–790 nm using an LED monochromator (SPG-120IR, Shimadzu Corporation, Kyoto, Japan) during surgery on the back table in the operating room. During this procedure, the operating room lights were turned off. The autofluorescence illumination of each parathyroid gland at each projected EWL was monitored and recorded by a commercially available NIR video camera system (LIGHTVISION, Shimadzu Corporation) in a sterile environment. This device visualizes and records NIR fluorescence illumination of 800–850 nm, even under optic visual white light. The EWL projection for this apparatus is 800 nm, which was not used for this study because the value was specifically adjusted to optimally monitor the fluorescence of ICG and not the autofluorescence of the parathyroid gland. The lights in the room were turned off for these measurements for obtaining a clear and distinct image. The autofluorescence intensity of the three normal parathyroid glands were monitored and recorded as video clip images. The autofluorescence illumination intensity was then measured using the ImageJ software (National Institutes of Health) (14) at each alternating EWL. A 3-mm diameter region of interest was defined for each sample to measure with the ImageJ, and the maximum autofluorescence intensity of each sample was normalized as one. The autofluorescence intensity curve of the three parathyroid glands were obtained to compare the curve form for each parathyroid gland to clarify the optimum EWL. Neither operative nor postoperative complications were reported throughout the procedure. A small proportion of the samples were all confirmed intraoperatively and postoperatively to be normal parathyroid gland by routine intraoperative rapid frozen pathologic examination and by postoperative pathologic examination with hematoxylin and eosin stain, performed by the pathologist of the authors' institution. For all patients, all remaining parathyroid glands that were transplantable were auto transplanted into the sternocleidomastoid muscle in an effort to maintain postoperative parathyroid function.

### Comparison of Auto Fluorescent Intensity and Images of the Parathyroid Gland With Surrounding Structures at Optimum EWL

A total of nine sets of parathyroid gland, thyroid gland, lymph nodes, and fat were collected from six consecutive patients who underwent thyroidectomy at the authors' institution from January–May 2020. All patients volunteered to undergo monitoring of the parathyroid glands, thyroid gland, lymph nodes, and fat by an NIR camera during thyroid surgery for measurements to compare the intensity of fluorescence. The criteria were applied except the availability of the samples and monitoring normal parathyroids, so findings of preoperative examinations concerning the parathyroids were all within normal. Each sample set consisted of a fragment or whole tissue of the parathyroid, thyroid, lymph node, and fat. Samples were pathologically identified. A total of eight sets were collected from five thyroid cancer patients who had total thyroidectomy, and one set was from one patient who had a left lobe thyroidectomy for adenomatous goiter. The samples were collected immediately following the resection of the thyroid organ. The samples were handled with sterile conditions. Fluorescent illumination was video imaged using the pde-neoII NIR fluorescence imager C10935-400 (Hamamatsu Photonics K.K., Hamamatsu, Japan) system on the back table in the operating room. The pde-neoII is an apparatus that projects 760-nm NIR EWL by LED and collects the fluorescence illumination at 830 nm. It is a commercially distributed device to visualize fluorescence using ICG dye for blood vessels and tissue perfusion and for the detection of the sentinel lymph node in early breast cancer surgery. This device was used because the projected EWL was close to the optimum EWL measured in the experiment described herein. The video images were captured, and ImageJ application was used to measure the actual fluorescence illumination of the collected samples. Each video image clip set consisted of the four different samples and aligned in one single image so relative illuminance intensity could be measured based on the comparison to the brightness ratio to the thyroid, which was normalized as one. Actual observation of the image was presented using the LIGHTVISION under the condition when the optimum EWL was projected and applied. The handset of the pde-neoII, which projects EWL of 760 nm, was used to project the optimum EWL to observe the extraction of the embedded parathyroid gland from the lower pole of the thyroid.

## Ethical Consent

All patients directly provided their consent either orally or by writing before surgery for the measurement of the surgical samples for fluorescent illumination by the attending surgeon. Moreover, standard surgical procedures were also explained. An institutional review board registration was not requested because all procedures were within the standard surgical procedures and did not affect the outcome of the therapy. All procedures were in accordance with the ethical standards of the institutional and/or national research committee and with the 1964 Helsinki Declaration and its later amendments or comparable ethical standards.

## Statistical Analysis

For statistical analysis, R version 3.6.3 was used. The Welch *t* test was performed to determine the statistical difference of the average illumination ratio between the four samples (*p* < 0.05).

## Results

### Measurement of the Optimum EWL for the Parathyroid Gland

**Figure 1:**
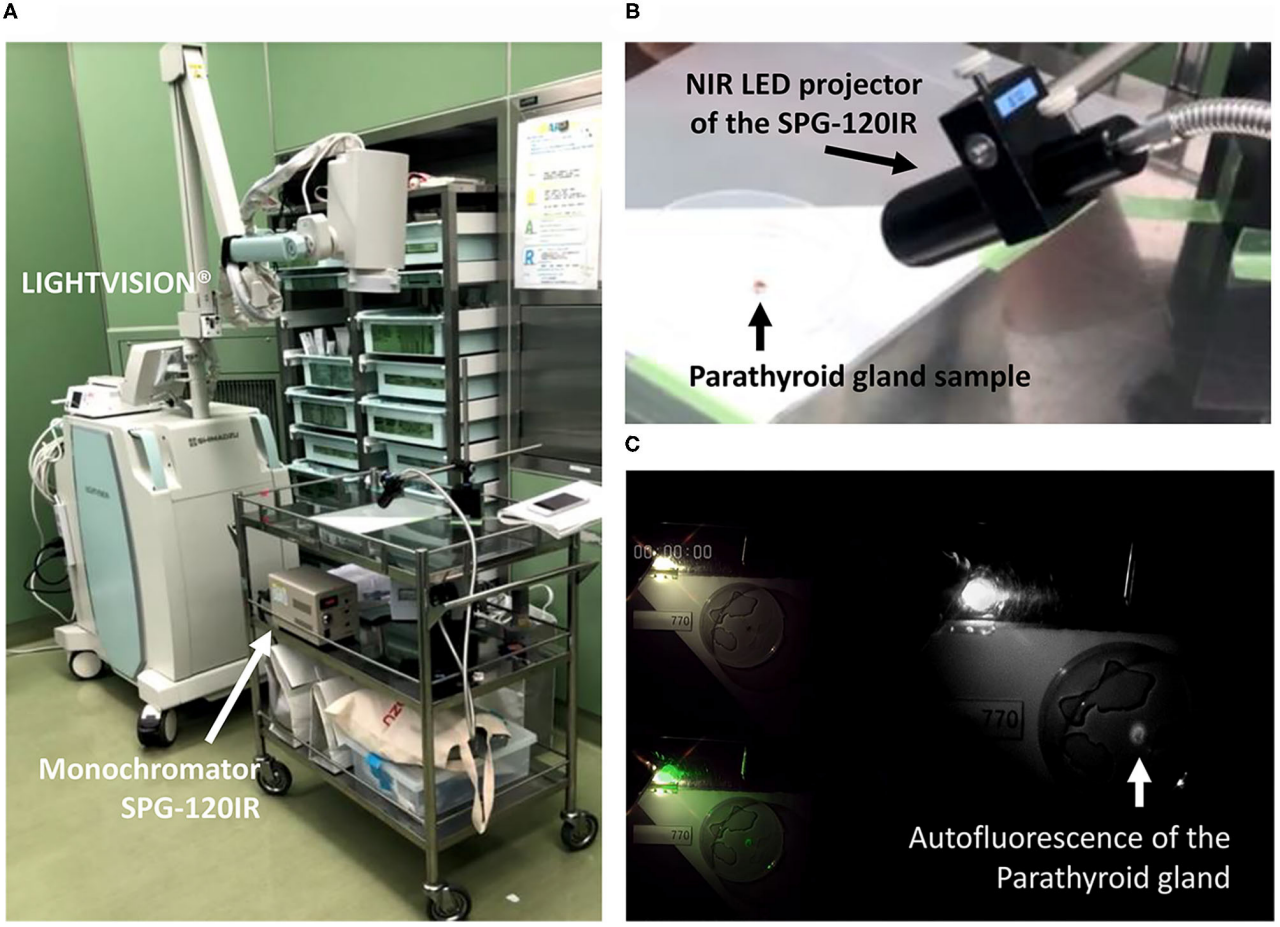
Device used to measure the EWL characteristics of the parathyroid gland**: (A)** the fluorescence illumination was recorded with the LIGHTVISION NIR camera; **(A,B)** NIR EWL was projected to the extracted parathyroid gland from the monochromator SPG-120IR; **(C)** illumination data from the video recorded image were analyzed using ImageJ; the image is lightened by penlight to show the sample and projected EWL nm. EWL, excitation wavelength; NIR, near infrared.

**Figure 2:**
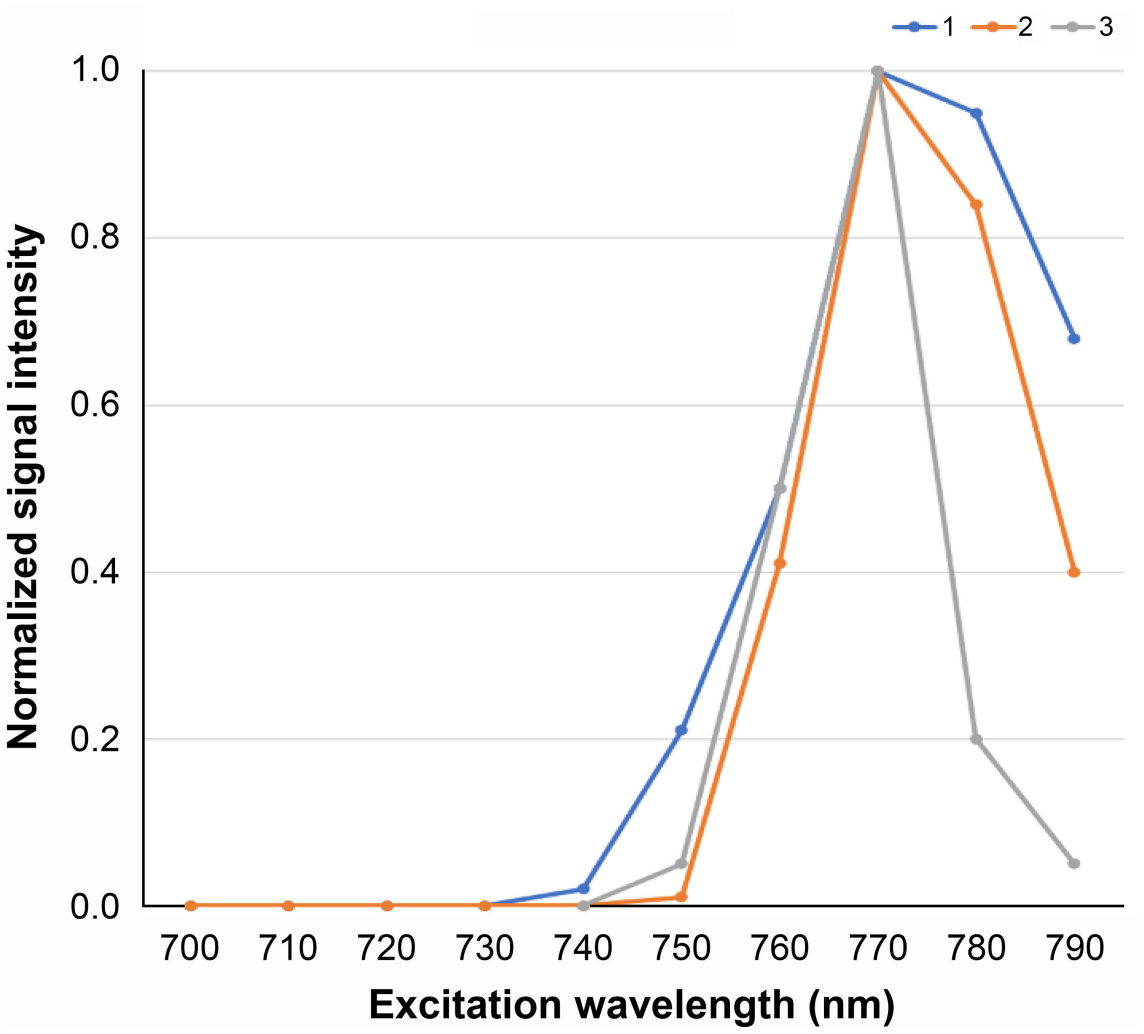
Autofluorescence intensity characteristics for the three parathyroid glands. The optimum excitation wavelength for the parathyroid gland to autofluorescence absorbance wavelength of 800–850 nm was estimated to be 770 nm.

The autofluorescence intensity of three pathologically proven normal parathyroid glands were monitored and recorded as video clips using the LIGHTVISION system as shown in Figure 1A. The LED monochromator (SPG-120IR) used in this experiment to project the altered EWL in order to search the optimum EWL is shown in Figure 1B. The monitor image of the LIGHVISION with the room lights turned off was video clipped to measure the fluorescence (Figure 1C). The intensity of the fluorescence illumination of the parathyroid gland was captured and each image was measured using the ImageJ. The peak measurement of each of the samples was normalized so the maximum value was one. The intensity characteristics for the three parathyroid glands were identical, and three samples were judged enough for the study (Figure 2). Measurement with 700 nm EWL at 10-nm intervals up to 790 nm EWL showed that the illumination intensity started to increase at 740 nm and peaked at 770 nm. Based on the measurements of these three samples, the optimum EWL under the LED light source for the parathyroid gland with autofluorescence absorbance wavelength of 800–850 nm was estimated to be 760–770 nm.

### Comparison of Auto Fluorescent Intensity and Images of the Parathyroid Gland With Surrounding Structures at Optimum EWL

**Figure 3:**
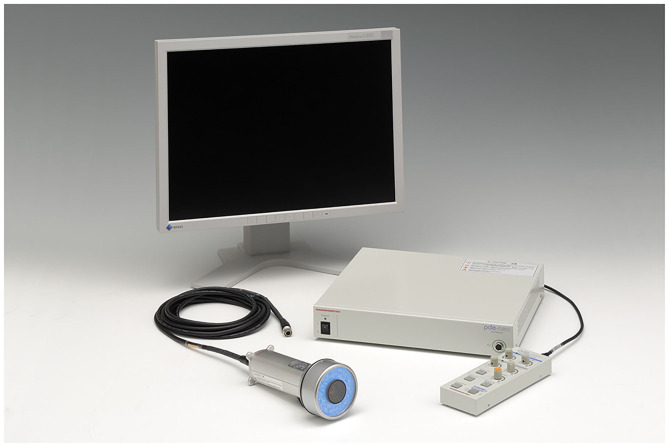
The pde-neoII NIR fluorescence imager C10935-400 used to monitor extracted samples. This device projects 760-nm NIR EWL by LED and collects the fluorescence illumination at 830 nm. This device is commercially sold to detect the sentinel lymph node in the breast cancer surgery with fluorescent light using indocyanine green. EWL, excitation wavelength; LED, light-emitting diode; NIR, near infrared.

**Figure 4:**
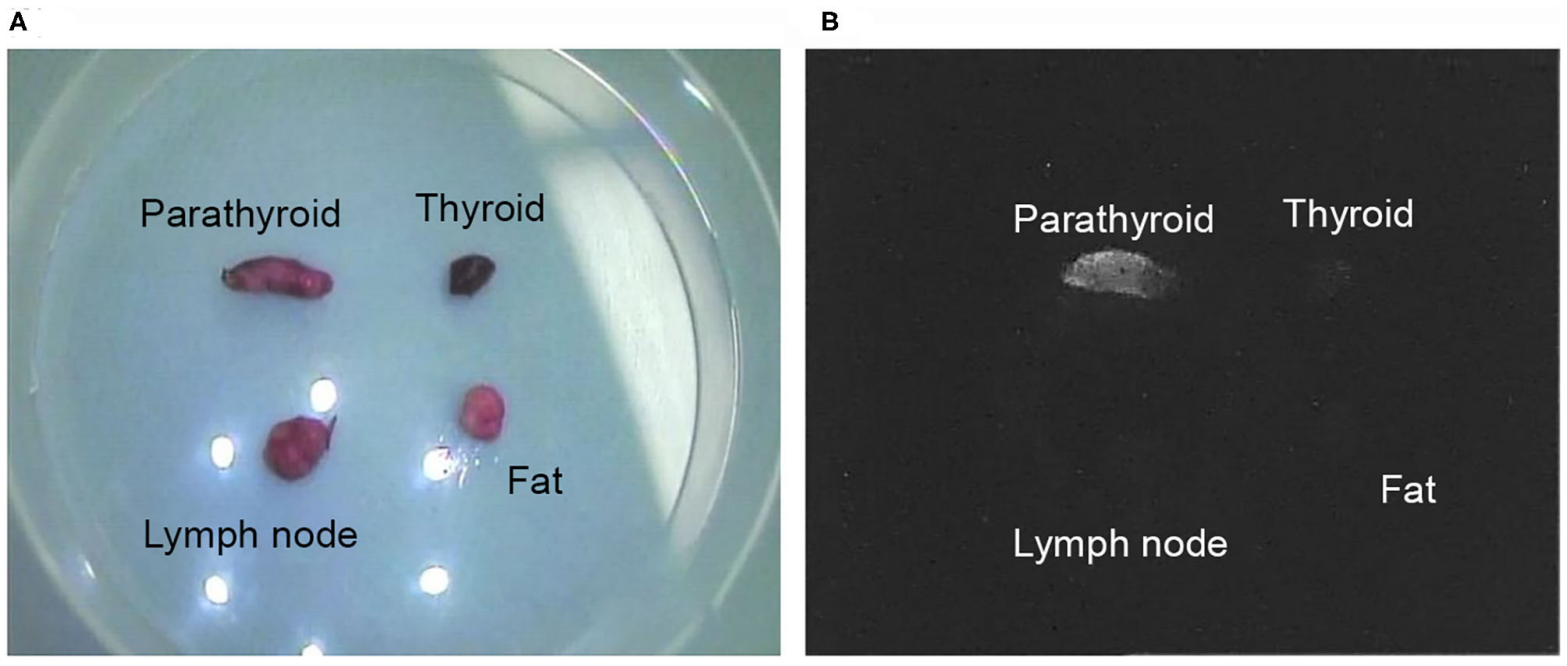
Video image sample clip of the collected material. One sample set image clip of the parathyroid gland, thyroid, lymph node, and fat tissue are shown in one view to compare the relative illumination intensity ratio: **(A)** the optic visual light view under white light; **(B)** and fluorescence view.

Fluorescent illumination for each sample sets were video imaged using the pde-neoII NIR fluorescence imager C10935-400 (Figure 3). Figure 4 shows of one image of the sample set that was recorded and captured with the pde-neoII NIR fluorescence imager. The video image of each set containing the four samples was the video clip captured, and the 3-mm diameter region of interest was defined to measure and compare the illumination intensity ratio.

**Figure 5:**
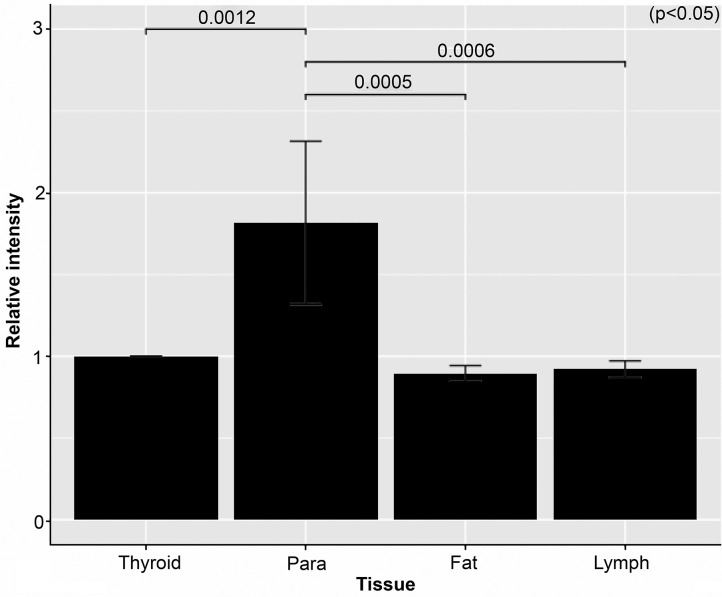
Graph of the relative intensity of each tissue. The average relative intensity for the nine sample sets of the parathyroid (Para), fat, and lymph node (Lymph) compared with the thyroid is shown. As shown parathyroid was almost 2-fold brighter.

**Figure 6:**
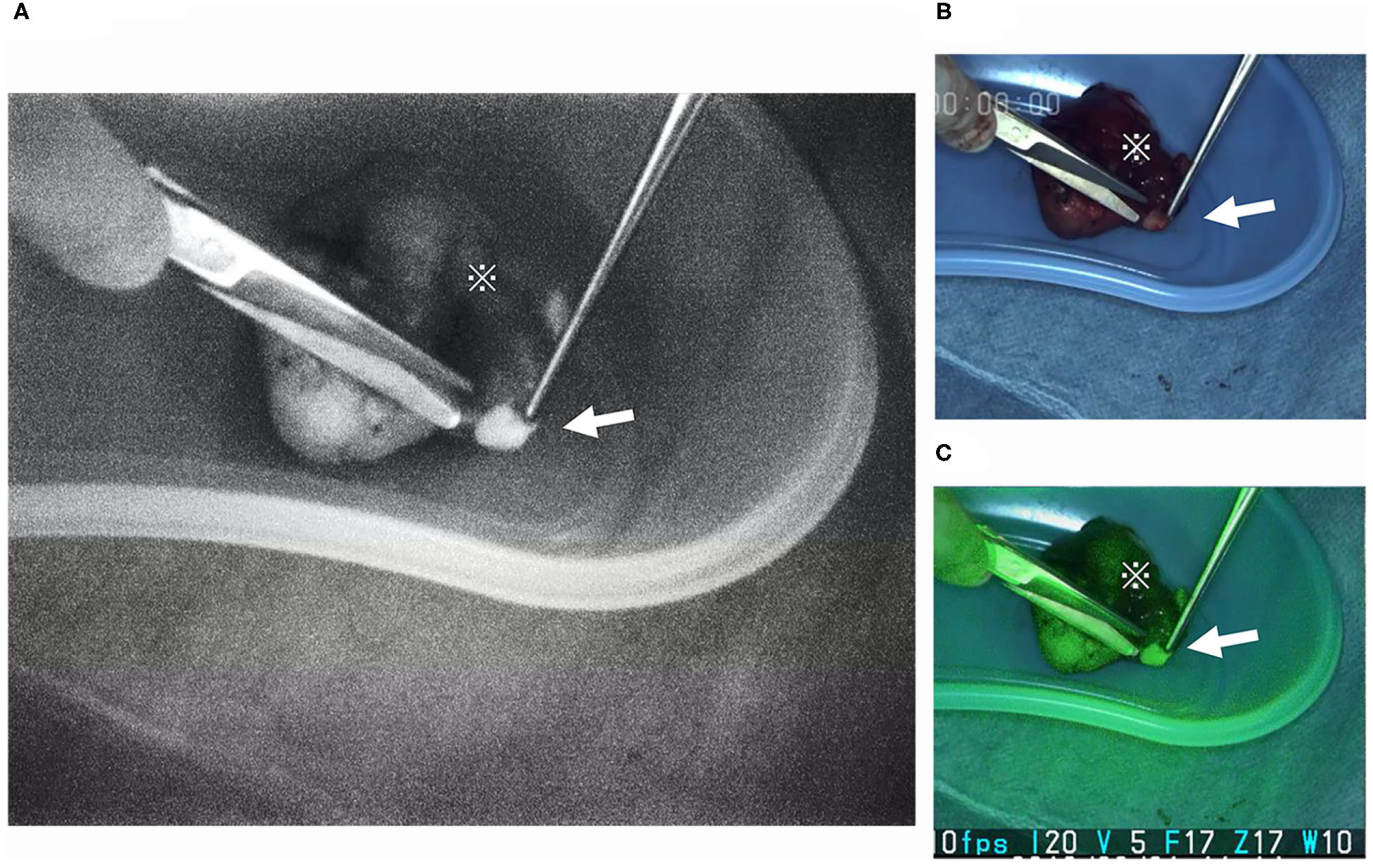
Extraction of the parathyroid gland at the back table in the operating room. The parathyroid gland (arrow) is often embedded with in the lower pole of the excised thyroid (※). The parathyroid gland is easily detected and located with the NIR camera. The projection source of the pde-neoII was used. The image is visualized using the LIGHTVISION NIR camera. This device enables three screens visualizing: **(A)** the NIR fluorescence, **(B)** the optic white light, and **(C)** the optic and modified NIR fluorescence screen visions projected simultaneously. NIR, near infrared.

Results for the measurements using the ImageJ application are given in Figure 5. With thyroid intensity measurements normalized to one, the average intensities were as follows: 1.82 for parathyroid gland, 0.82 for lymph nodes, and 0.89 for fat tissues. A statistically significant difference was observed on comparison between the fluorescence illuminance of the parathyroid and the other tissues (*p* < 0.05). The luminal intensity of the parathyroid gland was almost 2-fold greater compared with the surrounding structures of thyroid, lymph nodes, and fat. A video image clip of the detection and extraction of the inferior parathyroid gland located in the lower pole of the thyroid is shown in Figure 6. This image clip is taken by the LIGHTVISON under room light with the exposure EWL at 760 nm using the handheld projection source of the pde-neoII. A clear image and distinct detection of the parathyroid gland is observed located in the lower pole of the thyroid.

## Discussion

Identification of the parathyroid gland is essential when performing the surgical techniques of thyroidectomy and parathyroidectomy (15); however, detection is difficult because the parathyroid gland may vary in number and anatomic location (1). The unintended removal or injury of the parathyroid gland during thyroidectomy is reported to occur in up to 31% of cases. Especially when thyroidectomy with dissection of the pre- and paratracheal lymph nodes is the standard surgical procedure for the thyroid carcinoma, unintended removal of the parathyroid occur because the inferior parathyroid gland is often embedded within. This removal will result in hypoparathyroidism and complications such as postoperative hypocalcemia, for which daily administration of enormous amounts of calcium drugs are required lifelong, noted to occur in 5.5% of patients with postoperative hypocalcemia. To detect the parathyroid from the extracted surgical specimen, possible salvage for autotransplantation is important to prevent possible postoperative hypocalcemia. The detection of the parathyroid glands during surgery mainly relies on the visual skills of the trained surgeon. Intraoperative rapid frozen pathologic diagnosis is used to detect the parathyroid; however, the process of sampling of the gland involves the extraction of part of the gland and thus damage or devascularization cannot be avoided. Some institutions have the access to real-time measurement for the serum concentration of intact parathyroid hormone (16). A decrease in this serum concentration expresses the extraction or devascularization of the parathyroid glands; however, this information is not always useful for the preservation of an intact parathyroid gland. Classical diagnostic imaging such as ultrasonography and enhanced computed tomography can detect abnormally swollen parathyroid glands, but these modalities are typically only useful preoperatively, not as real-time techniques, and thus present difficulty in detecting normal-size glands. The use of technetium (^99m^Tc) sestamibi-guided surgery has been reported to detect abnormal parathyroid glands, but the efficacy was unreliable for detection of the normal glands (17). To meet the need for real-time detection of the parathyroid gland during surgery, the NIR device has been used since 2010 (6, 9). The electromagnetic wave NIR has a wavelength of ~700–2500 nm, which is slightly below the optically visual “red” light wave and is used for devices in daily living such as the television remote control unit. The vein authentication used for biometric identification, such as in automated teller machines, is another example of the application of an NIR device. In this application, the strong NIR absorption property of hemoglobin is used in which veins appear darker in contrast to the surrounding perimetry. In the medical field, this technique is applied in vascular access imaging devices used internationally in the hospital emergency departments. The use of the fluorescence property of the ICG to evaluate the efficacy of the skin flap, vessel anastomosis, and digestive tract anastomosis is now a common application of the technique in surgery (4, 5). Also, in 2005, Kitai reported using ICG to detect the sentinel lymph node for breast cancer surgery (3). For detection of the parathyroid glands, many promising reports have documented the utility of ICG (6–8). However, reported also noted that the blood flow of the surrounding thyroid may also reflux, and distinct differences in the illumination intensity changes between the tissues may be rendered.

This study focused on the auto fluorescent characteristics of the parathyroid gland under an LED light source to obtain simultaneous optic visual identification. The investigation aimed to improve the sensitivity and specificity of detection of the parathyroid gland, which have been reported as 94.1 and 80%, respectively, by De Leeuw et al. (12). With no agents such as ICG to inject, this approach was thought less invasive, much simpler, and more direct. The detection rates for ICG injection and autofluorescence of the parathyroid glands are reported to be the same, 95 vs. 98%, respectively. Compared with the ICG-fluorescing glands, however, an advantage of autofluorescence is that it can more frequently detect the parathyroid gland before recognition by the naked eye (8). In 2011, Paras et al. first reported detection of the peak autofluorescence for the parathyroid gland occurring at 820–830 nm. In their study a 785-nm diode laser was used as the NIR light source (9). Several clinical studies have followed this report of the successful use of the laser device (12, 13, 18, 19), although these laser light sources are not yet medically insured or permitted in Japan for clinical use. Thereafter, use of the NIR camera with LED lighting as a commercially available device was introduced and typically programmed arranged to detect ICG fluorescence (13). As previously noted, numerous studies of this approach to indicate the parathyroid gland have been conducted, but the success rates and the devices used have varied (12, 13, 19, 20). Focusing on the undefined optimum EWL for the parathyroid gland, the present study aimed to measure the optimum EWL of the surgically extracted parathyroid gland. Also, the NIR light source was LED-generated, which enabled visual confirmation of the object. The authors believe the definition of the optimum EWL will also lead to the development of an NIR camera with improved accuracy for the discovery of the parathyroid glands to prevent unintended parathyroidectomy during surgery.

It is critical to note that the mechanism behind the intrinsic autofluorescence of the parathyroid gland is yet unknown. The calcium-sensing receptor as the primary fluorophore candidate has been proposed (21). The receptor is present with high concentrations in the chief cells of the parathyroid gland, but the theory for autofluorescence has not yet been validated. Other potential fluorophores have been suggested such as secretory granules, pseudo-colloids, and porphyrin derivatives (18). The development of a microscopic device that can project the optimal EWL and that will potentially emit the fluorophore should also be beneficial in unraveling the mechanism for the intrinsic autofluorescence of the parathyroid gland.

One of the limitations of this study is the small sample size. A larger sample size may result in a more reliable conclusion; as stated above, identical optimum EWL of the three samples were considered sufficient to reach a conclusion. Another is that the measurements were made from surgically extracted samples. To observe the fluoresce illumination characteristics of the intact parathyroid gland, *in vivo* (*in situ*) unextracted measurements are required. Because blood flow with infrared-absorbing hemoglobin is abundant in the parathyroid as well as in the thyroid, the illumination may not be as bright compared with the extracted counterparts that were measured in this study. For comparison of the illumination ratio to the surrounding thyroid, lymph node, and fat, however, the use of an NIR camera device with the optimum EWL of 760–770 nm should be effective in detecting the parathyroid gland in contrast to the other structures. Also, the fluorescence visible to the naked eye should be sufficient and reliable for the operative methods in thyroidectomy, including extraction of the parathyroid gland for evaluation at the back table of the operating room as well as supplementary auto transplantation to the sternocleidomastoid muscle to retain parathyroid function because the parathyroid gland of the lower pole is often embedded within the excised thyroid or central neck compartment at lymphadenectomy. In such cases, if the extracted samples were compared, the 2-fold difference shown in this study is certainly compatible with these goals, and the NIR camera using the LED with optic vision was shown to be particularly useful (Figure 6). The clear and distinct detection of the parathyroid gland exposed at the optimum EWL demonstrates the importance of defining and application the optimum EWL.

To be able to identify visually the parathyroid gland during real-time surgery by using the NIR camera with the optimal EWL of 760–770 nm will surely benefit the outcomes for the surgical therapy by improving the detection rate of the parathyroid gland, even for the less-experienced surgeon. In addition, the ability to detect and acknowledge whether the parathyroid gland is preserved and intact, will be time saving, be less stressful for the surgeon, and will provide reassurance for the success of the surgical procedure. Finally, this approach may lead to less postoperative complications, such as hypocalcemia caused by hypoparathyroidism by unintended removal of the parathyroid glands and will also benefit the patient for the overall surgical outcomes.

## Conclusion

The optimum EWL for the autofluorescence of the parathyroid gland was measured at 760–770 nm. Comparing the fluorescence illumination between parathyroid gland with surrounding structures of the thyroid, lymph node, and fat showed a difference of a 2-fold greater illumination intensity with the use of the NIR camera with this EWL value. These LED-projected fluorescence images can be confirmed by the optic vision of the surgeon and video recorded. Although observed with extracted parathyroid gland material, translating these study data to detect the parathyroid gland with better accuracy using the NIR imaging should benefit clinical therapy by reducing complications and improving surgical outcomes. Also, this approach should benefit the development of a device to unravel the mystery of the mechanism for intrinsic autofluorescence of the parathyroid gland. Further research including *in situ* observation and clarification of the mechanism are essential along with characteristics of pathological parathyroid glands.

## Data Availability Statement

The raw data supporting the conclusions of this article will be made available by the authors, without undue reservation.

## Ethics Statement

Ethical review and approval was not required for the study on human participants in accordance with the local legislation and institutional requirements. The patients/participants provided their written informed consent to participate in this study.

## Author Contributions

Material preparation, data collection, and analysis were performed by IT, AzF, AsF, and HT. The first draft of the manuscript was written by IT. All authors contributed to the study conception and design, commented on previous versions of the manuscript, and read and approved the final manuscript.

## Conflict of Interest

Equipment used in this study were provided by Shimadzu Corporation and Hamamatsu Photonics K.K. The authors declare that the research was conducted in the absence of any commercial or financial relationships that could be construed as a potential conflict of interest.
